# The identification of a spontaneous 47, XX, +21/46, XY chimeric fetus with male genitalia

**DOI:** 10.1186/1471-2350-13-85

**Published:** 2012-09-20

**Authors:** Kuei-Fang Lee, Chun-Shuo Hsu, Pao-Lin Kuo, Jing-Liang Chen, Yuan-Hong Jiang, Ingrid Y Liu

**Affiliations:** 1 Laboratory for Cytogenetics, Center for Genetic Counseling, Buddhist Tzu Chi General Hospital, 707, Sec 3, Chunyang Rd, Hualien, 970, Taiwan; 2 Graduate Institute of Medical Sciences, Tzu Chi University, Hualien County, 970, Taiwan; 3 Department of Obstetrics & Gynecology, Buddhist Tzu Chi General Hospital, Dalin Branch, Chiayi County, 622, Taiwan; 4 Department of Medical Laboratory Science and Biotechnology, National Cheng Kung University, 1, University Road, Tainan City, 701, Taiwan; 5 Department of Urology, Buddhist Tzu Chi General Hospital, 707, Sec 3, Chunyang Rd, Hualien County, 970, Taiwan; 6 Department of Molecular Biology and Human Genetics, Tzu Chi University, 701, Sec 3, Chunyang Rd, Hualien, 970, Taiwan

**Keywords:** Chimerism, Trisomy 21, Sex chromosome, Fetus, Genitalia, Karyotype

## Abstract

**Background:**

Approximately 30 sex-chromosome discordant chimera cases have been reported to date, of which only four cases carried trisomy 21. Here, we present an additional case, an aborted fetus with a karyotype of 47,XX, +21/46,XY.

**Case presentation:**

Autopsy demonstrated that this fetus was normally developed and had male genitalia. Major characteristics of Down syndrome were not observed except an enlarged gap between the first and second toes. Karyotyping of tissues cultured from the fetus revealed the same chimeric chromosomal composition detected in the amniotic fluid but with a different ratio of [47,XX,+21] to [46,XY]. Further short tandem repeat analysis indicated a double paternal contribution and single maternal contribution to the fetus, with the additional chromosome 21 in the [47,XX,+21] cell lineage originating from the paternal side.

**Conclusion:**

We thus propose that this chimeric fetus was formed via the dispermic fertilization of a parthenogenetic ovum with one (Y) sperm and one (X,+21) sperm.

## Background

Sex-chromosome discordant chimeras (46,XX/46,XY) are extremely rare in humans; in fact, its incidence is undetermined [1]. Gartler *et al*. described the first case of sex-chromosome discordant chimera as well as a hermaphrodite (with ambiguous genitalia) formed via double fertilization in 1962 [2], and approximately 30 cases have been reported since [3]. There are especially few reported cases of chimerism involving coexisting normal and abnormal lineages that each carries a distinct sex chromosome complement. Only four cases of sex-chromosome discordant chimerism with trisomy 21 have been reported to date. One was a true hermaphrodite [4], one was a newborn infant with ambiguous genitalia [5], and other two cases had normal gonads [6,7]. Three cases harbored an extra chromosome 21 in their XY lineages [4-6], and one case had mosaicism in the XX lineage [7]. Here, we present the fifth case of sex-chromosome discord4nt chimerism, with trisomy 21 (47, XX + 21/46, XY) and male genitalia.

## Case presentation

A phenotypically normal 21-year-old female with an obstetric history of gravida 1 and para 0 (G1P0) requested an amniocentesis at the 18^th^ week of gestation because of anxiety. Although prenatal ultrasound examinations appeared normal, amniocentesis revealed a sex chromosome-discordant chimeric karyotype with trisomy 21 in the XX lineage (47,XX,+21/46,XY). After receiving genetic counselling, she and her non-consanguineous husband, a 29-year-old with acquired mutism caused by suspected viral infection at the age of 7 months, decided to terminate the pregnancy via induced vaginal delivery at the 21^st^ week. They agreed to donate their blood and the aborted fetus for further medical research.

## Methods

### Autopsy

The aborted fetus was 15.5 cm in body length and weighed 262 g. The head, trunk, and extremities appeared grossly normal. Autopsy was performed according to standard procedures. Tissues from brain, kidneys, skin, placenta, and cord blood were collected for karyotyping. DNA wa4 isolated directly from the brain for further genetic analysis.

### Karyotypig and preparation of genomic DNA

Karyotying was performed as standard protocol. Genomic DNA was purified according to the manual of DNA isolation kit produced by Gentra system,USA.

### Short-tandem-repeat genotyping

Short-tandem-repeat genotyping was performed according to the manual of Human Sex and Autosomal STR Mapping Set (GenePhile G-Plex Kit, GenePhile Bioscience Co., Ltd, Taiwan). A total of 16 STR markers were applied: AMEL amplifies different length of fragments from X and Y chromosome and the remaining 15 are autosomal markers, of which one is on chromosome 21 (D21S1437). Additional 3 STR markers specific to chromosome 21 were obtained from AmpFlSTR ® Identifiler ™ PCR Amplification Kit (Applied Biosystems, Foster City, CA, USA) (D21S11) and Bioscience Co., Ltd (D21S1436 and D21S1270). GenePhile G-Plex PCR Amplification Kit (GenePhile Bioscience Co., Ltd, Taiwan) was used for STR genotyping. Genotypes were scored using Gene Scan® and Genotyper® softwares (Applied Biosystems, USA) and were verified blindly by three technicians.

### Ethics statement

This research was performed abiding by the regulation of the institutional review board (IRB) case 098–82 of the Tzu-Chi general hospital. Written informed consent has been obtained from the parents for publication of this case report and accompanying images.

## Results

### Morphological examination of the fetus

**Figure 1:**
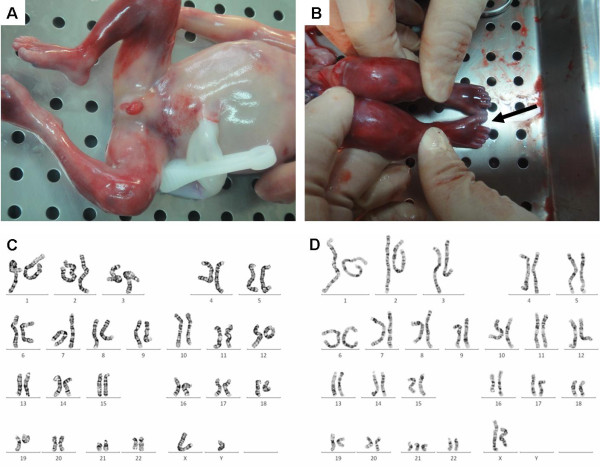
**Autopsy and karyotypes of the chimera fetus.** The aborted fetus exhibits normal external genitalia (**A**) and no characteristics typical of Down syndrome except the enlarged gap between the first and the second toes (**B**) was observed. The fetal karyotypic ratio of 46,XY (**C**) to 47,XX,+21 (**D**) in cortical cells was 11:9.

The morphologies of the fetal head, face, trunk, and extremities appeared to be grossly normal, as did the internal organs, including the heart, lungs, gastrointestinal, hepatobiliary, and genitourinary systems. External genitalia and gonads revealed normal male development, though bilateral cryptorchidism was observed at this embryonic stage (Figure 1A). Characteristics of Down syndrome were not obvious except for an enlarged gap between the first and the second toes (Figure 1B).

### Karyotypes

Chromosomal analyses of the parents revealed normal karyotypes (figures not shown). Karyotypes obtained from various cultured fetal tissues indicated the presence of sex-chromosome discordant trisomy 21 chimerism but with different ratios of [47,XX,+21] to [46,XY]. Twenty cells from the cortex and placenta and sixteen cells from the skin and kidney were analyzed. The ratio of [46,XY] (Figure 1C) to [47,XX, +21] (Figure 1D) was 11:9 in the cortex and placenta and 11:5 in the skin and kidney.

### STR analysis

**Figure 2:**
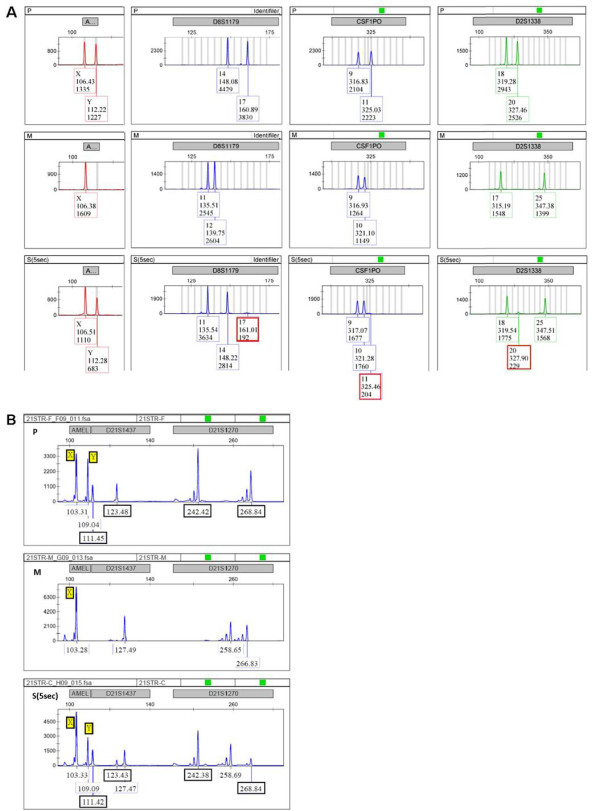
**STR analysis.** The sex chromosome-specific marker amelogenin (AMEL) (the first marker shown on A and B) indicates that the fetal sex chromosomes are disomic. The markers D2S1338, CSF1PO, and D8S1179 (**A**) demonstrate the presence of two heterozygous alleles from the father and one allele from the mother. The markers D21S1270 and D21S1437 (**B**) indicate that the fetus inherited two paternal and one maternal chromosome 21. (P: paternal; M: maternal; S: fetus).

To clarify mechanism of the chimera formation and identify the origin of the additional chromosome 21, we used 19 markers (Table 1) to perform STR analyses on samples from the parents and fetus. Only 6 of the 19 markers were informative (including AMEL); 4 of the informative markers localized to chromosomes Y, 2 (D2S1338), 5 (CSF1PO), 8 (D8S1179), and 2 localized to chromosome 21 (DS21S1270 and D21S1437). The sex chromosome-specific marker amelogenin (AMEL) indicated disomic sex chromosomes (Figure 2A). The autosomal markers D2S1338, CSF1PO, D8S1179 (Figure 2A) and chromosome 21 markers D21S1270, D21S1437 (Figure 2B) indicated that the fetus inherited two (heterozygous) alleles from the father and one allele from the mother. STR analyses identified a double paternal contribution (including chromosome 21) and one maternal contribution to the fetal genotype.

**Table 1:** Chromosome map position for STR markers used in the present report

**STR marker**	**Chr. Position**	**Father**		**Mother**		**Fetus**		
		**map position**		**map position**		**map position**		
D2S1338	2	319.28	327.46	315.19	347.38	319.54	347.51	327.90
TPOX	2	229.54	241.52	241.55		229.67	241.48	
D3S1358	3	128.06		123.98	127.99	128.15	124.02	
FGA	4	226.14	234.13	238.14	246.29	234.24	238.31	
D5S818	5	155.13		146.81	159.47	155.26	159.47	
CSF1PO	5	316.83	325.03	316.93	321.10	317.07	321.28	325.46
D7S820	7	278.62		270.62	278.77	270.71	278.77	
D8S1179	8	148.08	160.89	135.51	139.75	148.22	135.54	161.01
Tho1	11	170.87	182.80	182.87		170.87		182.80
vWA	12	178.41	186.30	178.50		178.54	186.43	
D13S317	13	216.40	220.42	224.49	232.40	216.58	232.40	
D16S539	16	271.91	275.88	275.91	283.97	271.93	276.03	
D18S51	18	294.27		290.16	307.19	294.41	307.26	
D19S433	19	121.71		121.68		121.72		
D21S11	21	203.82	207.78	217.67		203.93	217.74	
D21S1270	21	242.42	268.84	258.65	266.83	242.38	258.69	268.84
D21S1437	21	111.45	123.48	127.49		111.42	127.47	123.43
D21S1436	21	174.55	190.29	174.54		174.49	190.29	
								
AMEL	X		106.43		106.38		106.51	
	Y		112.22				112.28	

## Conclusion

**Figure 3:**
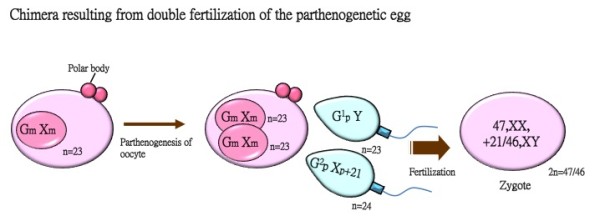
**The mechanism of the formation of the present chimeric case with trisomy 21.** This chimeric case could result from the fertilization of a parthenogenetic egg with two sperms, one (Y) sperm and one (X, +21) sperm.

Several mechanisms may be involved in the formation of an XX/XY chimera: 1) a tetragametic chimera results from the post-zygotic fusion of two distinct embryos; 2) a chimera results from the dispermic fertilization of an self –replicated oocyte or the second polar body; 3) an androgenetic chimera (isodisomic paternal cell lines) occurs when one normal zygote fuses with another zygote formed via one sperm fertilizing with an egg that is empty of genetic material (for a review, please see [1]). In addition, a post-zygotic diploidization and non-disjunction of chromosome 21 of a triploid is also possible. In cases of tetragametic chimeras, both double paternal and double maternal chromosome contributions would be detected. Recently, *in vitro* fertilization appears to increase the incidence of tetragametic chimeras [8,9] because standard practices necessitate the utilization of more than one embryo to increase success rates. Molecular analyses have been performed for 2 [4,7] of the 4 previously reported cases with sex chromosome discordant chimerism with trisomy 21. Both were tetragametic chimeras and revealed a paternal origin of the extra 21^st^ chromosome. Sex-chromosome discordant tetragametic chimera can also be fused from two abnormal cell linages. A stillborn male fetus with multiple congenital anomalies was identified to have a (47, XY, +21/47, XX, +12) karyotype [10]. In chimera cases that result from double fertilization of a self-replicated egg or the second polar body, a double paternal contribution and single maternal contribution would be detected. According to our STR analysis, this is the putative mechanism that led to the genotype of the present case. This fetus may have been formed from a parthenogenically-activated oocyte fertilized with two sperms with opposite sex chromosomes, one of which harbored an extra chromosome 21 (Figure 3). The possibility of double fertilization with the second polar body is considered very rare because there is a lack of evidence of crossing over events, which requires further analysis with more STR markers. In addition, the coexistence of both (47,XX,+21) cells and (46,XY) cells in the present case were detected in various tissues originating from different germ layers, indicating that the formation of this chimeric fetus occurred at an extremely early embryonic development stage. A sex-chromosome discordant chimerism with ambiguous genitalia and trisomy 14 in the XY cell linage (46, XX/47, XY, +14) was reported to form via similar mechanism [11].

The prenatal detection rate of 46,XX/46,XY from heterogeneous amniotic cells is approximately 0.24% [12]. Maternal cell contamination of the male fetal amniotic fluid accounts for the large majority of cases. Other cases of 46, XX/46, XY may result from laboratory error due to cross-contamination between two samples [3]. The death of a twin would not result in coexistence of XX and XY cells in the amniotic fluid because cells in dead tissues stop dividing and would not be detected by karyotyping [12]. Thus, it is only after excluding these possibilities that the prenatal diagnosis of chimerism or mosaicism may be considered [1]. Most sex-chromosome discordant chimeras (XX/XY) are diagnosed due to the presence of ambiguous genitalia at birth, and these individuals account for approximately 13% of true hermaphrodites. Phenotypic expression is highly variable for sex-chromosome discordant chimeras and may range from normal to ambiguous external genitalia. Those with ambiguous genitalia (hermaphrodites) may develop normally and even reproduce successfully [1]. Most sex-chromosome discordant chimerism cases demonstrate normal cognitive function. However, sexual ambiguity, infertility and the possibility of developing gonadoblastomas are major concerns in these individuals. At an early embryonic stage, chimeras with trisomy 21 appear to be grossly normal, as was observed in our case and the case that was reported by Hwa *et al.*[7]. However, it is difficult to predict whether such cases would develop normally at later stages. The phenotypic expressions of such cases are variable. One case was shown to exhibit multiple congenital defects and anencephaly [6], another presented with ambiguous genitalia [5], and one exhibited with ambiguous genitalia but no clinical features of Down syndrome [4]. Genetic counseling for sex-chromosome discordant chimeras with or without trisomy 21 can be extremely difficult. Informative counseling regarding cognitive function, sexual ambiguity, infertility, and the possibility of developing gonadoblastomas should be provided.

## Consent

This research was performed abiding by the regulation of the institutional review board (IRB) case 098–82 of the Tzu-Chi general hospital. Written informed consent has been obtained from the parents for publication of this case report and accompanying images.

## Competing interests

The authors declare that they have no competing interests.

## Authors’ contribution

K.-F. L carried out cytogenetic and molecular cytogenetic experiments. C.-S. H. carried out pedigree analysis, clinical examination and diagnosis of this family. J.-L. C. and Y.-H. J. performed anatomical examination and drafted “Patient and clinical examination” section. P.-L. K. performed STR analyses. I.Y.L. conceived of the study, and participated in its design, coordination and manuscript writing. All authors read and approved the final manuscript.

## Pre-publication history

The pre-publication history for this paper can be accessed here:

http://www.biomedcentral.com/1471-2350/13/85/prepub
